# Molecular characterization of *Cryptosporidium* spp. obtained from fecal samples of immunosuppressed patients from Brazil

**DOI:** 10.1590/0037-8682-0555-2021

**Published:** 2022-04-08

**Authors:** Flávia de Souza Cunha, Higor Wilson Jann, Jocemir Ronaldo Lugon, José Mauro Peralta, Regina Helena Saramago Peralta

**Affiliations:** 1 Universidade Federal Fluminense, Faculdade de Medicina, Departamento de Patologia, Niterói, RJ, Brasil.; 2 Universidade Federal Fluminense, Faculdade de Medicina, Departamento de Nefrologia, Niterói, RJ, Brasil.; 3 Universidade Federal do Rio de Janeiro, Instituto de Microbiologia Paulo de Góes, Departamento de Imunologia, Rio de Janeiro, RJ, Brasil.

**Keywords:** *Cryptosporidium* spp, Immunosuppressed populations, Kidney transplant, Genetic diversity

## Abstract

**Background:**

*Cryptosporidium* spp. are pathogenic protozoans that play an important role in developing diseases in the elderly, children, and immunosuppressed individuals.

**Methods:**

The objective of this study was to detect and genetically characterize *Cryptosporidium* spp. in kidney transplanted patients (n = 97 samples; group 1) and immunosuppressed individuals from an outpatient clinic suspected of having *Cryptosporidium* infection (n = 53 samples; group 2). All fecal samples were analyzed by parasitological stool examination, immunochromatographic test, and real-time polymerase chain reaction (real-time PCR). *Cryptosporidium*-positive samples were tested using nested PCR for the gp60 gene, followed by sequencing for subtype determination.

**Results:**

Parasitological examination was negative in all Group 1, and positive in four Group 2 samples. Real-time PCR revealed *Cryptosporidium* in 13 samples: four in Group 1 (three *C. hominis* and one *C. parvum*) and nine in Group 2 (seven *C. hominis*, one *C. parvum*, and one mixed *C. hominis/C. parvum*). The immunochromatographic test was reactive in 11 samples (four in Group 1 and seven in Group 2). All 11 *C. hominis* isolates were identified as subtype IbA10G2 and one *C. parvum* as subtype IIbA15G2R1. All *C. hominis* belonged to subtype IbA10G2, which is recognized as the most prevalent and pathogenic subtype.

**Conclusions:**

This study showed, for the first time, that the presence of *Cryptosporidium* subtypes is considered more virulent in Brazilian transplanted kidney patients.

## INTRODUCTION

*Cryptosporidium* spp. is an important protozoan related to gastrointestinal tract infections in various hosts^1^. This protozoan has recently been reclassified as gregarine^2^ based on excision and sequencing of the 18S rRNA gene^3^. The genus comprises at least 38 described species recognized worldwide for causing diarrhea in humans and animals^4^; however, *Cryptosporidium hominis* and *Cryptosporidium parvum* are the main species responsible for cryptosporidiosis in humans^5^. 

This parasite plays an essential role in the development of diseases in the elderly, children, and immunosuppressed individuals, such as patients infected with HIV, as well as those undergoing cancer therapy and transplantation^6^ ^,^ ^7^. In the transplantation group, immunosuppressive drugs induced a decrease in immunity, especially in the first three months post-transplantation^8^. 

Since different species of *Cryptosporidium* are morphologically and morphometrically identical, molecular methods have become important tools to distinguish these species, and they constitute fundamental strategies for understanding the transmission of *Cryptosporidium* infection^9^. These techniques could also be applied to *Cryptosporidium* subtype determination and, consequently, an important tool for epidemiological and transmission studies^10^. Laboratory diagnosis of cryptosporidiosis is generally made by direct examination of stool samples by optical microscopy. In addition, antigens or genetic material can be detected in this sample, and specific antibodies to *Cryptosporidium* spp. can be detected in serum samples^11^ ^,^ ^12^. However, only molecular methods have been able to differentiate *Cryptosporidium* species and improve their laboratory diagnosis^5^ ^,^ ^13^. 

Epidemiological studies in Brazil that define *Cryptosporidium* species and subtypes are scarce, and most of them have been applied to specific target populations^14^ ^,^ ^19^. However, to understand and define the prognosis and severity of *Cryptosporidium* infection, genotype and subtype determination, as well as clonality, could be important tools for future epidemiological studies^20^ ^,^ ^21^. Thus, the objective of this study was to characterize the genetic diversity of *Cryptosporidium* spp. obtained from fecal samples of patients undergoing kidney transplantation and from individuals from the general federal hospital service who fulfilled the criteria for *Cryptosporidium* detection.

## METHODS

### Sample collection from patients and parasitological exams by microscopy

This study was conducted in two different groups of patients. The first group consisted of patients from the Hospital São Francisco de Assis (HSF), Rio de Janeiro, Brazil, who underwent kidney transplantation (Group 1), while the second Group included all the samples that were entered into the Laboratory of Parasitology of Hospital Universitário Antônio Pedro (HUAP), Niterói, Rio de Janeiro, Brazil, with a request for *Cryptosporidium* and coccidia search (Group 2). 

For Group 1, stool samples were collected from the same individual at two time-points: the first collection was conducted in the pre-surgery period of the transplant or up to the first seven postoperative days, while the second collection was performed three months after transplantation, following the highest level of immunosuppression. A total of 72 patients participated in the survey; however, only 25 returned for the second collection. The patients had a mean age of 50 years (range,25-75 years), 45 were men (27 women). The standard protocol to induce immunosuppression in patients undergoing kidney transplantation includes the use of prednisone, everolimus, sirolimus, sodium mycophenolate, thymoglobulin, and tacrolimus. In most cases, 3 or 4 of these drugs were used. In the maintenance stage of immunosuppression, which will be performed for life, when possible, an association of three drugs is made, in general, using prednisone, tacrolimus, and one-third drug chosen considering the general conditions of the patients. The dosage was adjusted according to the immunosuppression stage. Group 2 consisted of adult patients, with a mean age of 42 years (range,21-69 years), including 33 men and 20 women. Based on the doctor´s information request, 38 patients were HIV-infected, and 10 had solid organ transplants for at least 2 years. In the other five patients, the request was from the outpatient clinic without precise information.

A total of 150 stool samples from patients in Group1 (n = 97) and Group 2 (n = 53) were processed for parasitological stool examination (PSE) using the Lutz spontaneous sedimentation method for general intestinal parasite diagnosis and a centrifuge sedimentation technique with two centrifugations of 2.000 x g/5 min, followed by fecal smears stained with the Safranin-Methylene Blue technique^22^ for identification of *Cryptosporidium* oocysts. One aliquot of each sample was stored at -20 °C until molecular characterization.

This study was conducted with the approval of the Ethical Review Committee for Research, Faculty of Medicine, Federal Fluminense University, CAAE 44050814.4.0000.5243, 07/06/2015.

### Immunochromatographic test for *Cryptosporidium* spp.

The feces were diluted in a buffer provided by the manufacturer, and the supernatants of these samples were used to perform the test using the RIDA® QUICK Cryptosporidium N1203 kit (R-Biopharm, AG, Germany), following the manufacturer's instructions.

### DNA extraction

The FastDNA^TM^ SPIN Kit for feces (MP Biomedicals) was used for total genomic DNA extraction from patient stool samples, following the manufacturer’s instructions. The samples were disrupted using an FP120 cell disruptor (MP Biomedicals). The DNA extracts were stored at -20ºC until use.

### TaqMan PCR assays

The TaqMan PCR procedure combines a duplex reaction to detect of *Cryptosporidium* spp. and *C. parvum* and a simple reaction for the detection of *C. hominis*, as described previously^18^ ^,^ ^23^. Assays were performed using a 7500 Real-Time PCR System (Life Technologies, Carlsbad, CA, USA). Primers (JVAF, JVAR, JVAGF, and JVAGR) and probes (JVAP 18S and JVAGP2) were added to the reaction following a methodology described before^18^ ^,^ ^23^. All assays included positive controls (*C. hominis* and *C. parvum*) and negative controls (DNA extracted from fecal samples negative for any parasites). The presence of inhibitory substances was analyzed through contamination of negative samples with 10fg of *Cryptosporidium* DNA and subsequently subjected to TaqMan PCR.

### Subtyping analysis 

*C. parvum* and *C. hominis* were subtyped by DNA sequencing of gp60 gene following the protocol described by Glaberman et al. (2001)^24^. Each sample was amplified at least three times using PCR. Primers AL3531 and AL3533 (840 bp) were used in the primary PCR, and primers AL3532 and LX0029 (440 bp) were used in the secondary PCR. NucleoSpin® Extract II kit (MACHEREY-NAGEL GmbH and Co. KG, Germany) was used to purify gp60 products according to the manufacturer's instructions. Sequencing was carried out in both directions using the sequencing services of the Instituto de Biofisica Carlos Chagas - Macromolecular metabolism laboratory - UFRJ, Rio de Janeiro, Brazil. All nucleotide sequences were aligned with the reference sequences retrieved from GenBank. The resulting sequences were edited and aligned using the BioEdit Sequence Alignment Editor 7.0.5.3. and MEGA 4.1.

## RESULTS

### Parasitological examination and Cryptosporidium *spp.* oocyst detection in fecal samples

From March 2015 to May 2018, a total of 97 samples from HSF-transplanted patients - first collection (n = 72) and second collection (n = 25) (Group 1) and 53 samples from patients requesting *Cryptosporidium* detection (Group 2) were obtained. In Group 2, most of them were HIV-positive patients who had diarrhea (n = 45), and only eight of them were patients who had undergone solid transplantation at HUAP a few years ago. Among these 150 samples, PSE was performed, and only six samples from Group 2 presented *Blastocystis* spp., while one of them also had *Entamoeba coli*. No helminth was found.

Patients in Group 1 did not present positive results for *Cryptosporidium* spp. by microscopy at any time of parasitological examination. However, *Cryptosporidium* spp. was identified in four fecal smears from Group 2 (Table 1). 

**TABLE 1:** *Cryptosporidium* spp. positive stool samples from transplanted patients and outpatient clinic.

Samples	PSE	Safranin staining	Real-time PCR	***Cryptosporidium* copro antigen detection**
**DTR5**	Negative	Negative	*C. hominis*	Reactive
**DTR9**	Negative	Negative	*C. parvum*	Reactive
**DTR38**	Negative	Negative	*C. hominis*	Reactive
**DTR43**	Negative	Negative	*C. hominis*	Reactive
**DHU9**	Negative	Negative	*C. hominis*	Reactive
**DHU11**	Negative	*Cryptosporidium* spp.	*C. hominis*	Reactive
**DHU12**	Negative	*Cryptosporidium* spp.	*C. hominis/C. parvum*	Reactive
**DHU19**	Negative	*Cryptosporidium* spp.	*C. hominis*	Reactive
**DHU34**	Negative	*Cryptosporidium* spp.	*C. hominis*	Reactive
**DHU35**	*Blastocystis* spp.*, E. coli*	Negative	*C. parvum*	Negative
**DHU36**	*Blastocystis* spp.	Negative	*C. hominis*	Reactive
**DHU37**	*Blastocystis* spp.	Negative	*C. hominis*	Reactive
**DHU38**	*Blastocystis* spp.	Negative	*C. hominis*	Negative

**PSE:** parasitological stool exams, Staining - Safranin-Methylene Blue, **DTR:** transplanted patients (group 1), all had non-diarrheal stool samples, DHU - 5 HIV-infected patients, 2 old solid organ transplant, and medical clinic ward - all had diarrheal stool samples.

### Immunochromatographic test for antigen detection of *Cryptosporidium spp.*

All 150 samples from Groups 1 and 2 were subjected to an immunochromatographic test, and 11 were reactive to the *Cryptosporidium* spp. antigen. Within the 72 samples analyzed in their first sample, four were reactive (5.5%) (Table 1). None of the 25 samples from post-transplant patients tested positive in this test. However, in 53 samples from Group 2, seven were reactive (13.2%), showing higher sensitivity than microscopic examination. 

### *Cryptosporidium* detection by real-time PCR

DNA was extracted from 150 samples and subjected to real time PCR. Amplification was observed in 13 (8.7%) samples. Among the samples from Group 1 in their first collection (n = 72), four were PCR-positive (5.5%), three were *C. hominis*, and one was *C. parvum* (Table 1). In all samples from the second collection three months after transplantation (n = 25), no amplification was observed for *Cryptosporidium* spp. In Group 2, amplification was in nine (16.9%). Of these, seven were amplified for *C. hominis*, one for *C. parvum*, and one for a mixed *C. parvum*/*C. hominis* infection.

### *Cryptosporidium* spp. subtyping by gp60 nested PCR analysis

gp60 nested PCR was performed using the same 13 previously described real-time PCR-positive samples. Of these, 11 (84.6%) were amplified for gp60 and sequenced to define the subtype. Two samples that did not amplify the gp60 gene were the same as those that did not react for the immunochromatographic test. Sequence analysis revealed subtype IbA10G2 for *C. hominis* in all 10 samples obtained and subtype IIaA15G2R1 in one of *C. parvum*.

## DISCUSSION

In the present study, *Cryptosporidium* parasites were detected, and their genetic diversity was evaluated in clinical specimens obtained from immunosuppressed patients from two hospitals in Rio de Janeiro. In Brazil, laboratories do not use *Cryptosporidium* spp. detection as a routine diagnostic examination^15^ ^,^ ^18^ ^,^ ^25^, resulting in a lack of information on species and circulating subtypes^16^ ^,^ ^18^. According to the literature, information about enteric parasites in transplant patients is scarce, and most studies refer to parasites other than *Cryptosporidium*. There is a growing concern with this universe of patients, including hemodialysis patients, mainly in relation to the transmission routes of these parasites, which are environmental, especially in the water used for hemodialysis. Genotyping for the definition of species and subtypes has only been performed in a few studies, and none are related to these patients’ universe^15^ ^,^ ^18^ ^,^ ^19^ ^,^ ^26^ ^-^ ^29^.

In the present study, two parasitological methods were used: centrifugal sedimentation with methylene blue safranin staining for *Cryptosporidium* spp. and the spontaneous sedimentation method to detect other parasites. The results showed that few parasites were found using this methodology, even in some immunosuppressed patients with diarrhea. The convenient management of these patients could partly explain the findings. For example, Group 1 received nitazoxanide treatment during the pre-transplant period. Nitazoxanide is the only drug indicated for cases of diarrhea caused by *Cryptosporidium* spp. by the Food and Drug Administration (FDA) and is described on the website of the Centers for Disease Control and Prevention - CDC^30^. However, these agencies warn that the efficiency in patients with HIV and immunodeficiency may be low, and that patients with AIDS should receive treatment for longer periods (28 days). Even after treatment before transplantation, four cases were detected by rapid testing and PCR, indicating the importance of using laboratory tests with high sensitivity for groups at an elevated risk of developing cryptosporidiosis. However, PSE is known to have low sensitivity in detecting most enteric protozoa because of intermittent cyst release. The high number of negative PSE results for *Cryptosporidium* spp. may be explained by the need to eliminate many oocysts (50,000 to 500,000 oocysts per gram of stool). In addition, according to Huber et al. (2004)^31^, in the stool smear technique, reduced amounts of fecal specimens are examined, and examination of only two slides with stained fecal material is insufficient to detect *Cryptosporidium* spp. Thus, in apparently healthy subjects or those with antiparasitic treatment that eliminates few stool oocysts, this methodology would not be indicated. A study by Pacheco et al. (2013)^32^ showed that Safranin staining has a lower efficiency than Ziehl-Neelsen staining and modified auramine staining, which could partially explain the low sensitivity observed. However, this staining method has been implemented in the Parasitology Laboratory at HUAP for many years, with satisfactory results in the detection of coccidia, promoting a more homogeneous oocyst staining.

Several studies have investigated the incidence of opportunistic enteric parasites in immunosuppressed populations, particularly in people with HIV/AIDS. However, information regarding *Cryptosporidium* infection in kidney failure and transplant patients remains scant^33^. The fact that *Cryptosporidium* spp. are the most prevalent parasites in this population reinforces the need for its detection in routine laboratories using sensitive and specific methods^8^ ^,^ ^34^ ^-^ ^36^. Although microscopy is most commonly used to diagnose cryptosporidiosis in Brazil, it can only be performed by trained personnel. However, this method showed low sensitivity and did not allow for species or genotype identification as well as the available immunological methods. Thus, molecular methods, such as PCR, are required to detect small numbers of oocysts^5^, providing additional microscopic information and assisting epidemiologists in the surveillance of infectious agents and determination of sources of infection and virulence of these strains^15^ ^,^ ^18^ ^,^ ^37^ ^-^ ^40^.

The immunochromatographic test used as a qualitative method for detecting *Cryptosporidium* spp. in feces is efficient, fast, simple, and is not dependent on the presence of intact organisms (oocysts, cysts, or trophozoites) in stool samples. These fast, visual diagnostic methods are easy to implement and do not require sophisticated equipment or experienced staff to conduct them. The choice of diagnostic test should be based on two fundamental assumptions: sensitivity and specificity. Immunochromatographic tests that include monoclonal antibodies for detecting *Cryptosporidium* spp. have been evaluated in comparison with microscopy and PCR as reference techniques^41^. Studies carried out with the Rida®Quick immunochromatographic test showed 80-97% sensitivity in the diagnosis of *Cryptosporidium* spp. and *Giardia lamblia* ^41^ ^,^ ^42^. In our study, there was 85% agreement between the immunochromatographic test and real-time PCR, with only two samples differing. These samples were also negative in the other tests used, except for real-time PCR, and were obtained from patients without diarrhea. Stool immunoassays provide appropriate sensitivities and specificities with clinically relevant cost-effectiveness by providing rapid results for emergency situations in immunosuppressed transplant recipients, outbreaks, sample screening, and massive trials in endemic areas. It was observed that the sensitivity increased for parasite detection when molecular techniques were used, which was also observed in our group’s study^17^ ^,^ ^18^.

The presence of *C. hominis* was predominant in the analyzed samples, corroborating the literature findings that reported high infectivity for *C. hominis* in humans^41^ ^,^ ^42^. However, affirming that *C. hominis* is prevalent in Brazil is premature because the number of studies conducted in a human population is small, and all were developed in urban areas that favor anthroponotic transmission. The persistence of this infection in urban areas can be attributed to a set of reservoirs and sources of environmental contamination, as indicated by the detection of oocysts in healthy individuals, domestic and wild animals, and sewage and river water samples.

The gp60 gene, when analyzed for the *C. hominis* genotype, revealed a single subtype, IbA10G2. This same subtype was previously described by our group^18^ when immunosuppressed patients living in Rio de Janeiro were studied. The subtype IbA10G2 is also the most reported subtype in Latin America, although there are only a few studies^18^ ^,^ ^45^ ^,^ ^46^. In Argentina, within the same line of study, in addition to this more frequent subtype, another subtype of *C. hominis*, IeA11G3T3, was described. This subtype is also considered one of the most frequent subtypes in developing countries. The subtype IbA10G2 is cosmopolitan, present in most countries where this analysis was carried out, including Portugal, England, the United States, Peru, and Mexico. It is the most frequent subtype, is reported to be associated with increased infectivity and virulence and is present in different outbreaks worldwide^38^ ^,^ ^44^ ^,^ ^47^. Other subtypes such as IbA12G3, IbA13G3, IbA16G2, IbA19G2, IbA20G2, and IbA21G2 are restricted to certain regions. 

The only *C. parvum* detected in this study belonged to subtype IIaA15G2R1 and was different from our previous study, in which subtype IIcA5G3 was found to be anthroponotic^44^ ^,^ ^48^. This subtype predominates in calves and humans in different parts of the world^20^. The subtype IIa family has a high zoonotic potential, and it has also been described previously in a study carried out in calves from three farms in Rio de Janeiro state; however, subtype IIaA15G2R1 was not detected in this study^49^. In Latin America, subtype IIcA5G3 has been described in samples from Colombia, Jamaica, and Peru, whereas IIaA15G2R1 has only been described in Mexico^45^ ^,^ ^46^ ^,^ ^50^ ^,^ ^51^.

The global expansion of some virulent subtypes of *C. hominis* and *C. parvum*, such as IbA10G2 and IIaA15G1R1, presents great challenges in the identification and investigation of cryptosporidiosis outbreaks. Knowledge of the mechanisms underlying these subtypes is gradually being elucidated with several phenotypic and genotypic characteristics fundamental to understanding the epidemiology of cryptosporidiosis in industrialized and developing populations. New generation sequencing and genomic comparison are increasingly used for to characterize parasites isolated from humans and animals^43^ ^,^ ^52^.

This study reported for the first time, in Brazil, the presence and molecular characterization of *Cryptosporidium* in solid organ transplant patients and the subtype considered to be the most virulent among those already described. Despite the limited number of *Cryptosporidium-positive* samples for epidemiological conclusions in the kidney transplant patient group, the results highlight the need for further studies using different organ transplant recipients. Furthermore, these results reinforce the need to implement a more precise cryptosporidiosis diagnosis and prepare laboratories to determine the correct genotype or subtypes of *Cryptosporidium* spp. to improve clinical and epidemiological studies.
